# Attitude and Level of COVID-19 Vaccination among Women in Reproductive Age during the Fourth Pandemic Wave: A Cross-Sectional Study in Poland

**DOI:** 10.3390/ijerph19116872

**Published:** 2022-06-04

**Authors:** Jolanta Lis-Kuberka, Marta Berghausen-Mazur, Magdalena Orczyk-Pawiłowicz

**Affiliations:** 1 Department of Biochemistry and Immunochemistry, Division of Chemistry and Immunochemistry, Wroclaw Medical University, M. Skłodowskiej-Curie 48/50, 50-369 Wroclaw, Poland; 2 Department of Nursing and Obstetrics, Wroclaw Medical University, Bartla 5, 51-618 Wroclaw, Poland; marta.berghausen-mazur@umw.edu.pl

**Keywords:** COVID-19 vaccination, SARS-CoV-2 infections, benefits of vaccination, pregnancy, immune protection, public health, anxiety, specific anti-SARS-CoV-2 antibodies

## Abstract

COVID-19 vaccination, apart from the sanitary regime, is the most efficient strategy to limit the spread of the SARS-CoV-2 virus and significantly reduce the severity of the disease following infection. A cross-sectional survey was conducted during the fourth wave of the COVID-19 pandemic among pregnant Polish women and women who have already given birth to evaluate the level and attitude to vaccination. Briefly, 1196 women (256 pregnant and 940 mothers) participated in the study; 68.0% of pregnant women and 66.2% of mothers declared that they had received COVID-19 vaccination. The most frequently stated reasons not to get vaccinated were possible adverse effects on the mother, fetus or breastfed child, post-vaccination complications and limited scientific evidence on the safety of the COVID-19 vaccine. The identified predictors of avoiding COVID-19 vaccination are young age, residing in small cities or rural areas, cohabitation, low anxiety level regarding SARS-CoV-2 infection, and little knowledge concerning maternal vaccine-induced immune protection delivered to offspring. Despite the unlimited access to COVID-19 vaccination, the declared level of vaccination is worryingly low. The knowledge concerning the benefits of vaccination to mothers and their offspring is not satisfactory and requires urgent educational action, particularly among young women living outside big cities and single motherhood.

## 1. Introduction

The worldwide COVID-19 pandemic continues to rapidly spread across the globe, with substantial morbidity and mortality [1,2]. New variants of the SARS-CoV-2 virus are a critical public health challenge. The sex-disaggregated data on clinical characteristics and outcomes of hospitalized patients with COVID-19 suggest that men are more likely to have poor outcomes than women [3,4]. Nevertheless, there are indications that, from an economic and social point of view, the disease could disproportionately burden women [5]. These concerns are much more valid in relation to women in reproductive age, whose COVID-19 infection may result in serious clinical consequences to the health of both the mother and the child [6].

During the third wave of the pandemic, after the introduction of COVID-19 vaccines in Poland, the willingness to receive the COVID-19 vaccine was declared by 55% [7]; however, (in mid-February 2021) only 5.34% of the Polish population was fully vaccinated. During the fourth pandemic wave, 54.7% of the Polish population was vaccinated against COVID-19 (data on 12 December 2021) [8], and it ranks 23rd in the European Union in this respect. Although Poland has one of the highest death rates from COVID-19 in the world, unfortunately, at the beginning of the fifth wave of the pandemic, the percentage of vaccinated Poles did not increase significantly (56.96% of vaccinated, data on 30 January 2022). So far, there are no detailed data on the vaccination level of pregnant women and mothers who have already given birth in Poland, although the World Health Organization (WHO) [9] and public health institutions, including the Polish Neonatal Society, the Polish Society of Vaccinology, and the Lactation Science Center, recommend vaccination against COVID-19 with the mRNA preparation of Pfizer/BioNTech (Comirnaty) to pregnant and breastfeeding women [10,11].

Due to the pregnancy-related physiological changes in the immune, cardiovascular and respiratory systems, expectant mothers are in the group at higher risk of respiratory complications related to viral infections, including COVID-19. Pregnant women suffering from COVID-19 infection are at a higher risk for pregnancy-induced hypertension, thromboembolism, and finally preterm birth [12]. Moreover, pregnant women, in comparison to aged-matched non-pregnant patients, have an increased risk of intensive care unit admission, invasive ventilation, need for extracorporeal membrane oxygenation (ECMO), and finally, death [13,14,15,16,17]; however, the severity and clinical picture for the general population infected with SARS-CoV-2 and for infected pregnant women are at a similar level [18].

The mother’s immune system is crucial in the proper development and completion of a pregnancy [19]. Placental transfer of maternal immunoglobulin G (IgG) to the fetus is an important mechanism that provides protection to the child after delivery, while a baby’s immune system is immature [20,21,22,23]. The knowledge concerning the presence and level of SARS-CoV-2-specific Igs in blood and/or breast milk of pregnant and lactating women increases significantly with the successive waves of the pandemic [14,24,25,26,27,28]. The milk produced by mothers suffering from SARS-CoV-2 infection contains anti-SARS-CoV-2 specific antibodies, namely SIgA/IgA, IgG, and SIgM/IgM [29,30]. Transfer of neutralizing antibodies as a result of the maternal response to COVID-19 infection and/or vaccination may be crucial for neonates and infants, who, compared to older children, have a much more severe course of COVID-19 infection [23,31,32].

Unlike the early waves of the COVID-19 pandemic, in the fourth wave, the age of those infected fell sharply, including infants and young children. In addition, children below 2 years are at the greatest risk of developing acute symptoms [33] as well as long-term consequences, including PIMS-TS (pediatric inflammatory syndrome temporally associated with SARS-CoV-2) and MIS-C (multisystem inflammatory syndrome related to COVID-19). Unfortunately, there are currently no available vaccines for children under 5 years of age. In light of the above, the only effective and specific protection of newborns with an immature immune system is specific antibodies against SARS-CoV-2 that can be delivered during fetal development through the placenta, provided that the mother has been vaccinated before or during pregnancy and by the convalescent mother. Moreover, the protection of newborns and infants can also be continued after birth by consumption of milk produced by the vaccinated and/or convalescent mothers. Moreover, at present, it is very hard to pinpoint the health implications of the next wave of the pandemic for the health and wellbeing of pregnant women, mothers, and their babies, as well as the reproductive rate. Nevertheless, taking into account the data from 2020, namely the significant decrease in the birth rate of about 1.8% and in-hospital fatality rate of about 8.3%, the trend is not optimistic [34,35,36].

Considering the above, the aim of this study was to evaluate the vaccination level, attitude, and state of knowledge regarding COVID-19 vaccination during the fourth wave of the pandemic in Poland among pregnant women and women who have already given birth. Currently, there is a lack of reliable data that estimate women’s COVID-19 vaccination level during pregnancy and the postpartum period, although that group constitutes a large part of the population. Knowledge concerning the possible impact of sociodemographic and obstetric variables on the main concerns of receiving a COVID-19 vaccination during pregnancy and postpartum among Polish mothers is also lacking. Indication of areas with an insufficient level of knowledge will allow the construction of dedicated educational programs that can be implemented in this group to improve awareness of the benefits of vaccination, which in turn should translate into an increase in the vaccination level.

## 2. Materials and Methods

### 2.1. Study Design

**Figure 1:**
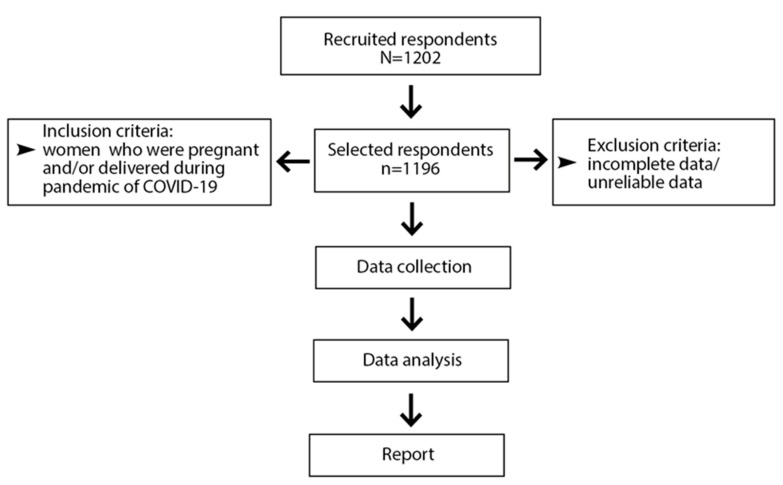
Flow chart of respondents included in the study.

As a research instrument, a questionnaire (https://forms.gle/ETxdQEWUvTfoC5VR9 (accessed from 15 November 2021 to 13 December 2021)) based on the research in the field was used. The survey was carried out during the fourth wave of the COVID-19 pandemic in Poland and addressed women who were pregnant or delivered a baby during the pandemic, i.e., from March 2020 to December 2021. Among the recruited responders, six women did not fully complete the questionnaire or provided unreliable data, probably due to inattention in completing the online survey (incomplete data/unreliable data) and were excluded from the analysis (Figure 1).

### 2.2. Questionnaire Development

An online, observational, nationwide, and cross-sectional questionnaire to evaluate COVID-19 vaccination among pregnant women and mothers who have already given birth was adopted. As a research tool, an anonymous online survey assessing the level of vaccinated women against COVID-19 as well as attitudes and knowledge concerning the benefits of maternal vaccination for the fetus and newborn was used. The final questionnaire was divided into three main sections: (i) sociodemographic and (ii) obstetric variables, and (iii) knowledge, attitude, and level of COVID-19 vaccination.

The questionnaire was initially tested and the feedback from 5 respondents was used to modify the final version of the online survey. The respondents were asked to answer open, single, and multiple-choice questions. The reliability of the questionnaire was estimated by determining Cronbach’s alpha value, which obtained a level 0.56 (from 0.30 to 0.62).

### 2.3. Data Source and Study Population

The study was conducted on the basis of a questionnaire approved by the Ethics Committee at Wrocław Medical University (No. KB-356/21). The women were recruited from 15 November 2021 to 13 December 2021 using the online survey. The questionnaire was administered using Google Forms, and potential respondents were recruited for the study through various parenting communities that stay in touch via Facebook. The randomly selected discussion groups, identified via Facebook, concerned the topics of maternity in the perinatal and postnatal period, such as breastfeeding and parenthood, and were visited by parents, especially mothers, who were sharing their experiences in the field of preparation for childbirth and natural feeding.

Taking into account the total number of births (344,000) from July 2020 to June 2021, and the level of vaccination of the Polish population with at least one dose of the COVID-19 vaccine (53.9%), the minimal estimated sample size was calculated using TIBCO STATISTICA ver. 13.3 (StatSoft, Inc., Tulsa, OK, USA) as 381 participants, with a confidence interval of 5% (absolute ± %) and a level of confidence of 95% (alpha = 0.05).

### 2.4. Data

#### 2.4.1. Sociodemographic Data

The sociodemographic data such as age and pre-pregnancy body mass index (BMI) were reported by the women participating in the study and collected as continuous variables. In the next step of data analysis, the ages of the participants were divided into five groups, namely: 18–25, 26–30, 31–35, 36–40, and ≥41 years, while BMIs (calculated based on the body weight and height provided by respondents and classified according to the WHO guidelines [37]) were categorized as follows: (1) underweight (BMI below 18.5 kg/m^2^), (2) normal weight (BMI ranging from 18.50 to 24.9 kg/m^2^), (3) overweight (BMI ranging from 25.0 to 29.9 kg/m^2^), (4) class 1 obesity (BMI ranging from 30 to 34.9 kg/m^2^), (5) class 2 obesity (BMI ranging from 35 to 39.9 kg/m^2^), and (6) class 3 obesity (BMI above 40 kg/m^2^).

The education level (university, high school, primary, and vocational), marital status (married, cohabiting, single parent, and divorced), and the place of the respondents’ residence (>100,000 inhabitants, 10,000–100,000 inhabitants, <10,000 inhabitants, and rural area) were determined.

#### 2.4.2. Obstetric Data

The obstetric data included: current obstetric state (pregnancy, a woman who gave birth to a child), mode of delivery (vaginal birth, elective, or emergency cesarean section), the course of pregnancy (with or without complications; yes, no), difficulties related to becoming pregnant (yes, no), the use of modern techniques supporting procreation (yes, no), and miscarriage in the past (yes, no).

#### 2.4.3. Assessment of Women’s Knowledge Level concerning COVID-19 Vaccination

The details concerning the assessment of knowledge levels were adapted from previous studies [38,39]. The following evaluation scale of grading was used: poor (<60%), moderate (60–79%), and detailed (80–100%).

#### 2.4.4. Women’s Knowledge and Attitude concerning SARS-CoV-2 Infections and COVID Vaccination

The questions in this section of the survey included: an assessment of the level of fear of contracting COVID-19 in the perinatal period (from 1 to 5, where 1 is equivalent to the statement ‘I was definitely not afraid’, and 5 is equivalent to the statement ‘I was definitely afraid’) (Were you afraid of SARS-CoV-2 infection during pregnancy?), and whether they had a COVID-19 infection (Did you suffer from COVID-19 during pregnancy or lactation?).

Moreover, the respondents were asked about their knowledge concerning the possibility of virus transmission through breast milk (is SARS-CoV-2 transmitted through breast milk?), placental transport of specific antibodies against SARS-CoV-2 to the fetus after vaccination of pregnant women (do you think that immunity achieved after COVID-19 vaccination might provide immune protection to the fetus and newborn (placental transfer)?) and protection of the newborn and infants through breastfeeding (do you think that immunity achieved after COVID-19 vaccination might be transferred with human milk to the newborns/infants?, and does breastfeeding protect children from COVID-19?).

#### 2.4.5. COVID-19 Vaccine Preference and Attitude

In this section of the survey, respondents who confirmed that they were vaccinated were asked with what preparation of the COVID-19 vaccine they were vaccinated (Pfizer/BioNTech, Moderna, AstraZeneca, or Johnson & Johnson), the main reason why they decided to get vaccinated and finally, the women were asked to qualify the level of concern related to the occurrence of complications after vaccination against COVID-19 (where 1 is equivalent to the statement ‘I was definitely not afraid’, and 5 is equivalent to the statement ‘I was definitely afraid’).

The unvaccinated women were asked to state the main reason why they did not get vaccinated and to qualify the level of concern related to the occurrence of complications after vaccination against COVID-19 (where 1 is equivalent to the statement ‘I was definitely not afraid’, and 5 is equivalent to the statement ‘I was definitely afraid’).

### 2.5. Statistical Analysis

The statistical analysis was performed with TIBCO STATISTICA ver. 13.3 (StatSoft, Inc., Tulsa, OK, USA). The manuscript contains continuous and categorical variables. The continuous variables of the analyzed parameters, namely the age and BMI of respondents (age, BMI), are shown as the median and the interquartile range, whereas the categorical variables are given as frequency and percentage (% (n/N)).

The Shapiro–Wilk test was used to evaluate the normality of the data distribution in relation to the variables. Due to the abnormal distribution of the data, the chi-squared test was used to evaluate the differences between the groups in terms of categorical variables. Additionally, the Kruskal–Wallis test was used for the continuous variables. A two-tailed *p*-value lower than 0.05 was regarded as significant. Multivariate logistic regression was used to determine the predictors of COVID-19 vaccination among women of reproductive age, which showed *p* < 0.05, using the odds ratio (OR). The level of confidence, according to the standards, was set at 95%, with a probability level indicated as statistically significant for *p* < 0.05. For building a multivariate logistic regression model, the COVID-19 Vaccination dataset contained 1196 observations and 10 variables, which were considered important (*p* < 0.05) during the 1st stage of analysis. During the creation of the multivariate logistic regression model, no variable selection method was used (“all effects” were selected), and the following parameters were obtained: R2 Nagelkerke 0.587, β coefficient *p* < 0.05, and *p*-value for Hosmer–Lemeshow’s test 0.324 (*p* > 0.05).

Moreover, the stratified analyses by vaccination and birth status (before or after giving birth to a child) were performed.

## 3. Results

### 3.1. Sociodemographic and Obstetric Data

The median age of the women participating in the study was 31.0 years, with an interquartile range of 28.0–34.0 years. Most of the women (765/1196; 64.0%) had a normal pre-pregnancy BMI and underweight, overweight, and obese women constituted 7.0%, 18.3%, and 10.7% of the total analyzed cohort, respectively. Mothers living in cities with a population of >100,000 (632/1196; 52.8%) constituted the most numerous group, while 18.9%, 6.4%, and 21.9% of the women reported that they lived in cities with a population ranging from 10,000 to 100,000, below 10,000, and in a rural area, respectively. More than three-fourths of participants (943/1196; 78.8%) reported having higher education. Married women were dominant in the analyzed group (959/1196; 80.2%), and single parenthood and divorced status were reported by only 1.9% of the respondents (23/1196) (Table 1).

Among all the women who completed the online survey, the highest percentage of respondents were mothers after delivery, 78.6% (940/1196), and more than half of mothers (528/940; 56.2%) reported that the pregnancy ended with vaginal delivery. Respondents who were currently pregnant represented 21.4% (256/1196) (Table 1).

The majority of respondents declared that they had no difficulties in conceiving a child (918/1196; 76.8%), although less than a tenth of them (7.8%; 93/1196) used medically assisted procreation, and nearly a quarter (21.7%; 259/1196) of the analyzed cohort had experienced a miscarriage in the past (Table 1).

### 3.2. Sociodemographic and Obstetric Data in Relation to COVID-19 Vaccination

In the present study, 66.5% (796/1196) of the women reported COVID-19 vaccination. The analysis of sociodemographic variables in relation to COVID-19 vaccination among women (*p* < 0.001), resided in big cities (*p* < 0.001), had higher education (*p* < 0.001), and were married (*p* < 0.001) (Table 2). In contrast, pre-pregnancy BMI did not differ significantly between the analyzed groups (*p* = 0.95).

On the other hand, the analysis of obstetric data in relation to COVID-19 vaccination among women (vaccinated, unvaccinated) showed no significant differences for: currently obstetric state (*p* = 0.35), mode of delivery (*p* = 0.11), complications during pregnancy (*p* = 0.63), difficulties in conceiving a child (*p* = 0.56), the use of modern techniques supporting procreation (*p* = 0.34), and miscarriage in the past (*p* = 0.69) (Table 2).

The analysis in relation to the obstetric status of women (pregnant and women after delivery) additionally revealed significant differences in the unvaccinated group with regards to body mass index (BMI) (*p* < 0.001), in the vaccinated group with regards to the course of pregnancy (physiological or with complications) (*p* < 0.006) and in both groups, namely vaccinated and unvaccinated, with regards to the place of residence (*p* < 0.005 and *p* < 0.001, respectively) (Table 3).

### 3.3. Women’s Knowledge concerning COVID-19 Vaccination

Among women vaccinated against COVID-19, 55.2% (440/796) were definitely afraid of SARS-CoV-2 infection during the perinatal period, while only 4.9% (39/796) stated that they were definitely not afraid of infection caused by the SARS-CoV-2 virus. In contrast, in the group of unvaccinated women, only 22.5% (90/400) reported that they were definitely afraid and 21.4% (86/400) did not fear SARS-CoV-2 infection, respectively (*p* < 0.001) (Table 4). The majority of the analyzed cohort reported that they did not suffer from COVID-19 infection, but the level was significantly higher (*p* < 0.001) in the vaccinated, 79.8% (635/796), compared to the unvaccinated, 63.0% (252/400), group.

Both vaccinated and unvaccinated respondents were familiar with the fact that COVID-19 is not transmitted through breast milk and represent a moderate level of knowledge in this area, but the level was significantly higher for the vaccinated than for the unvaccinated group, 75.3% (599/796) and 62.0% (248/400) (*p <* 0.001), respectively (Table 4).

The knowledge concerning delivery of maternal antibodies to the fetus by placental transport was significantly different; namely, vaccinated mothers showed detailed (88.1% (701/796)), in contrast to unvaccinated women who represented a poor level of knowledge (28.5% (114/400) (*p* < 0.001). A similar trend was observed for delivering maternal antibodies to newborns and infants through breast milk. A high level of knowledge was shown by vaccinated respondents independently of the way of immune system activation for the synthesis of specific antibodies, e.g., due to SARS-CoV-2 infection or as a response to vaccination, namely 72.7% (579/796) and 79.6% (634/796), respectively. In contrast, in the unvaccinated group, women showed a significantly lower level of knowledge (45.0% (180/400), and 24.5% (98/400)) (*p* < 0.001 and *p* < 0.001, respectively) (Table 4).

In addition, the anxiety and the knowledge levels concerning protection against COVID-19 were related to the obstetric state. Vaccinated pregnant women reported a significantly lower (*p* < 0.001) level (29.3% (51/174) of concern about the possibility of contracting SARS-CoV-2 in comparison to vaccinated women who have already become mothers (59.3% (369/622)). Similarly, a significantly lower (*p* < 0.001) proportion of pregnant respondents (60.9% (106/174)) were aware that SARS-CoV-2 is not transmitted through breast milk in relation to vaccinated mothers (79.3% (493/622)). Moreover, vaccinated women who have already become mothers revealed a significantly higher level of knowledge in regard to antibodies generated as a result of vaccination (82.8% (515/622)) or by COVID-19 infection (74.9% (466/622)) in comparison to vaccinated women during pregnancy (68.4% (119/174)) and 64.9% (113/174), respectively) (*p* < 0.001 and *p* < 0.001, respectively) (Table 5).

### 3.4. Women’s Knowledge concerning COVID-19 Vaccination in Relation to Sociodemographic and Obstetric Variables

Statistically significant differences were found between women’s knowledge concerning COVID-19 vaccination in relation to age (significant differences for four out of the six analyzed questions) but not to the BMI of the women who participated in the study (Supplementary Materials Table S1).

Additionally, the women’s knowledge concerning details related to COVID-19 vaccination was dependent on sociodemographic characteristics such as the place of residence, education, and the marital status of the respondents (Supplementary Materials Table S1). The COVID-19 knowledge level was higher for vaccinated women residing in big cities (significant differences for five out of the six analyzed questions) (*p* < 0.05), having a university degree (significant differences for all analyzed questions) (*p* < 0.05), and being married (significant differences for four out of the six analyzed questions) (*p* < 0.05).

Among obstetric data, the mode of delivery was statistically significant for three out of the six analyzed questions concerning attitudes of women toward vaccination against COVID-19 (*p* < 0.05). The course of pregnancy (normal or with complications) was statistically significant only for one question about the transfer of antibodies generated after vaccination with mother’s milk to the breastfed child (*p* = 0.03), while obstetric variables such as difficulties in conceiving a child and miscarriage in the past revealed a statistically significant correlation with the question concerning suffering from COVID-19 during pregnancy (*p* = 0.02 and *p* = 0.04, respectively) (Supplementary Materials Table S2).

### 3.5. COVID-19 Vaccine Preference and Attitude

**Figure 2:**
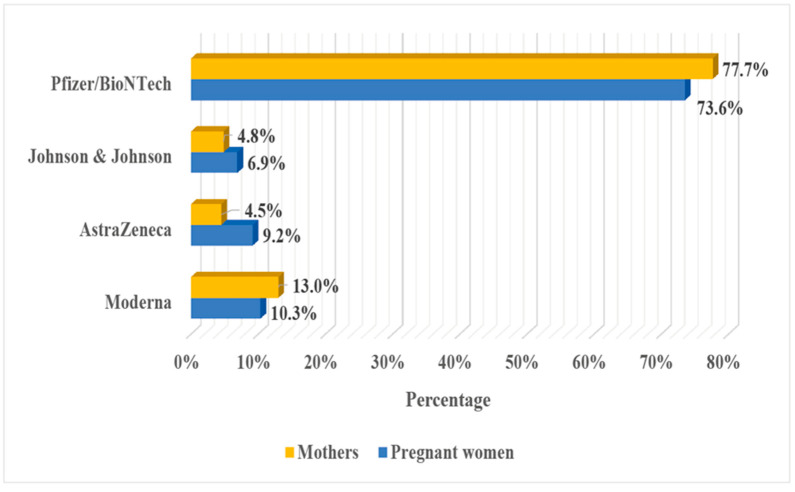
COVID-19 vaccine.

Among vaccinated respondents, the largest group constituted women who have been vaccinated with products of Pfizer/BioNTech (77.7% of pregnant women and 73.6% of women who had already given birth). The second group was women who received the Moderna vaccine (10.3% of pregnant women and 13.0% of mothers), while vaccines provided by AstraZeneca were received by 9.2% (16/174) of pregnant women and 4.5% (28/622) of mothers, while the Johnson & Johnson vaccine was received by 6.9% (12/174) and 4.8% (30/622) of vaccinated women during and after pregnancy, respectively (Figure 2).

**Figure 3:**
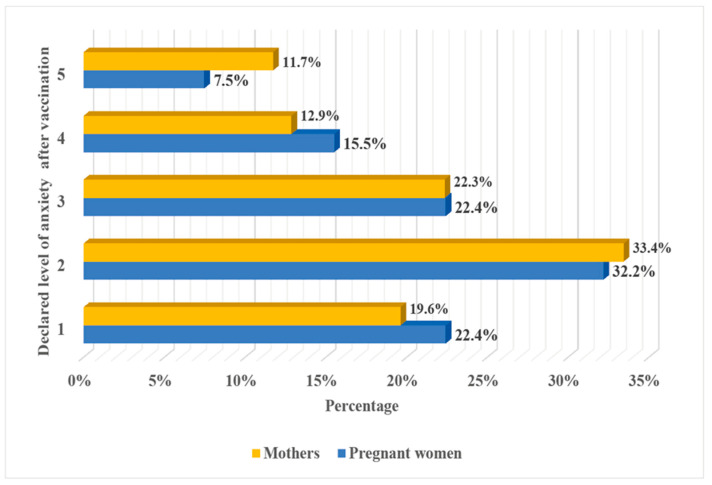
Declared level of anxiety of women after COVID-19 vaccination. Definitely not afraid—1 point on a five-point scale of fear. Definitely afraid—5 points on a five-point scale of fear.

In our study, 22.4% (39/174) of pregnant women and 19.6% (122/622) of women who had already given birth reported that they definitely did not fear (1 point on a five-point scale of fear) post-vaccination complications. In contrast, 11.7% (13/174) of vaccinated pregnant and 7.5% (73/622) of vaccinated mothers estimated their anxiety as 5 (definitely feared) on a five-point scale of fear (Figure 3), although the level of perceived fear regarding possible post-vaccination unwanted reactions did not differ significantly (chi^2^ 3.59, *p* = 0.46) between the analyzed groups of women in our cohort.

### 3.6. Respondents’ Reasons for COVID-19 Vaccination

Among the vaccinated cohort for both subgroups, the most frequently mentioned reason for COVID-19 vaccination was the overall protection against the severe course of the coronavirus infection, indicated by 60.3% (105/174) of pregnant women and by 62.4% (388/622) of mothers, respectively (Supplementary Materials Figure S1). As the main reason for vaccination, a higher risk of getting seriously ill from COVID-19, particularly during the perinatal period, was reported by 62.1% (108/174) of pregnant women, but only by 31.2% (194/622) of women who had already given birth. The COVID-19 vaccination during pregnancy as the reason for providing protection of the child during the first period after birth was reported by 40.8% (71/174) of pregnant women and 23.2% (144/622) of mothers, respectively. Detailed questions in this regard included immune protection due to the transfer of specific antibodies to the fetus (due to the placental transport) and breastfed child (due to the maternal milk). The protection of the fetus was indicated by 54.0% (94/174) of pregnant women and 28.1% (175/622) of mothers, while the protection of the child, due to the maternal milk, was reported by 20.1% (35/174) and 57.2% (356/622) of women during and after pregnancy, respectively. COVID-19 vaccination due to existing comorbidities was declared by 5.6% of pregnant women and 2.9% of mothers, respectively (Supplementary Materials Figure S1).

In our analyzed cohort, the respondents’ reasons for COVID-19 vaccination differed significantly (chi^2^ 105.37, *p* < 0.001) between the vaccinated pregnant women and vaccinated mothers.

### 3.7. Respondents’ Reasons to Avoid COVID-19 Vaccination

The avoidance of COVID-19 vaccination as the reason for possible overall post-vaccination complications was indicated by 73.2% (60/82) and 69.9% (222/318) of women during and after pregnancy, respectively (Supplementary Materials Figure S2).

In the area of detailed knowledge, as the most frequent reason to avoid COVID-19 vaccination among unvaccinated pregnant respondents, the possibility of adverse effects of vaccination on the developing fetus (81.7%; 67/82)) was reported. In contrast, in the subgroup of women who had already given birth, that reason was indicated by 55.7% (177/318) of respondents. The second and third most frequent reasons to not take the COVID-19 vaccination were possible adverse effects of vaccination on the mother and breastfed child, which were indicated by 61.0% (50/82) and 26.8% (22/82) women during pregnancy, and 60.7% (193/318) and (54.4%; 173/318) of women who had already given birth, respectively. Additionally, the pregnant respondents and mothers, as the reason not to take the COVID-19 vaccination, mentioned the presence of comorbidities (9.8% (8/82) and 14.2% (45/318), respectively) (Supplementary Materials Figure S2).

The most frequently self-reported reason to avoid COVID-19 vaccination indicated by women during and after pregnancy was the lack of detailed studies confirming the safety of the vaccine (2.4% (2/82) and 6.3% (20/318), respectively) (Supplementary Materials Figure S2). Other reasons prompting women to avoid COVID-19 vaccination include convalescent status, medical contraindications for vaccination, and the lack of trust (I do not believe in it), which were reported by 8.5% of pregnant women and 6.0% of women who had already given birth.

The factors mentioned by respondents as the main reason to avoid COVID-19 vaccination differed significantly (chi^2^ 21.02, *p* < 0.002) between the analyzed groups of unvaccinated women.

### 3.8. Predictors of a Lack of COVID-19 Vaccination among Women

For the analyzed cohort of women, the multivariate analysis identified that the respondents from small cities and rural areas avoided COVID-19 vaccination (OR = 2.06; 95% CI = 1.05–4.02 and OR = 1.89; 95% CI = 1.25–2.87). The COVID-19 vaccination was much lower for women in cohabitation than for married women (OR = 1.76; 95% CI = 1.15–2.70). Similarly, answering “Yes” on the following question: “Is SARS-CoV-2 transmitted through breastfeeding?” is a significant predictor of the lack of COVID-19 vaccination (OR = 2.32; 95% CI = 1.15–4.70). Moreover, the statement “No” or “I do not know” to the questions “Do you think that immunity achieved after COVID-19 vaccination might provide immune protection to the fetus and newborn (placental transfer)?” also significantly predicted the lack of COVID-19 vaccination (OR = 23.86; 95% CI = 12.99–43.84 and OR = 10.77; 95% CI = 7.20–16.10, respectively).

In contrast, the age of respondents is a weak predictor (OR = 0.96; 95% CI = 0.92–1.00) of opponents of COVID-19 vaccination among the analyzed cohort of women (Table 6).

## 4. Discussion

Based on a detailed search of the most valuable scientific databases, namely, PubMed, Scopus, and Web of Science, the attitude and level of COVID-19 vaccination among Polish women of reproductive age have not been analyzed before. In this study, an attempt was made to fill the gap with missing data for women in the perinatal period. Obtaining such detailed knowledge is necessary for the effective promotion of vaccination against COVID-19 in this population group, especially since the successive variants of the SARS-CoV-2 virus are responsible for the increase in the incidence of COVID-19 in the youngest age group. The detailed analysis of data collected during the fourth wave of the pandemic allows for the identification of barriers to receiving vaccines by women during pregnancy and mothers, namely the young women residing in medium-sized or small cities and rural areas. Our results are in line with sociodemographic variables, which differentiate between vaccinated and unvaccinated cohorts mentioned as important factors by other working groups [40,41,42,43,44,45].

The first study concerning the attitude of pregnant women to COVID-19 vaccination, when the preparation was not yet available, was carried out during the first wave of the pandemic in 2020 on the female population of the reproductive age living in Switzerland and showed that only 29.7% of pregnant women and 38.6% of lactating mothers declared a willingness to accept the vaccine against COVID-19 when the vaccine became available [40]. As the pandemic has unfolded, attitudes towards COVID-19 vaccination have changed [41,42,43]. At the end of 2020, when the vaccine was approved, the declared acceptance rate of COVID-19 vaccination was relatively high, namely 52.0% of pregnant women and 73.4% of mothers, but differed among analyzed populations [43]. Surprisingly, the intention to receive the vaccine was generally lower in Australia, Russia, and the United States than in India, the Philippines, and Latin America [43].

In early 2021 when the mass COVID-19 vaccination started but long-term studies on the COVID-19 vaccine were not yet available, only 28.2% of Italian women expressed a willingness to receive the COVID-19 vaccine during pregnancy [41]. In contrast, in the same period, but for a larger cohort, the attitudes and beliefs about COVID-19 vaccine acceptance among American women of reproductive age showed that pregnant respondents had a significantly higher rate of vaccine acceptance (44.3%) [42]. On the other hand, the willingness to receive the COVID-19 vaccine declared by pregnant women was lower than that declared by women during the postpartum period, namely 55.2%. A similar low acceptance rate to receive vaccination was indicated based on a cohort of Turkish pregnant women; namely, during January 2021, only 37% declared willingness to receive the vaccine if it is recommended for pregnant women, but the acceptance of vaccination was higher during the first semester in comparison to the subsequent trimesters [15].

Our study was carried out from 15 November to 13 December during the most dramatic period in Poland, when the number of deaths due to the COVID-19 was the highest since the beginning of the pandemic. In parallel, a significant increase in hospitalization of children under the age of two was recorded. Based on the survey, vaccination was declared by 68.0% (174/256) of pregnant respondents and 66.2% (622/940) of women who had already given birth. Surprisingly and optimistically, data turned out to be higher than the level of vaccination for the adult Polish population during the fourth and fifth waves of the pandemic, namely 56.96% and 57.7%, according to data provided by WHO, but it is one of the lowest in Europe [8].

Among 796 vaccinated women who took part in the survey, more than half (55.3%) declared the highest level of anxiety against SARS-CoV-2 infection, while this level of fear against SARS-CoV-2 infection in the group of unvaccinated women was reported by only 22.5% of respondents. The anxiety levels regarding a possible infection caused by SARS-CoV-2 have an impact on the decision concerning taking the COVID-19 vaccine. Similar conclusions were also presented by Schaal and coworkers [44]. Moreover, our findings are in line with the attitude regarding general vaccine acceptance and uptake presented by pregnant women in high-income countries; namely, women who evaluate the possible risk of infection at the highest level were more likely to receive vaccination [46].

The assessment of women’s knowledge level in the field of COVID-19 vaccination, including the transmission of the SARS-CoV-2 virus through breast milk, passive transport of specific anti-SARS-CoV-2 antibodies generated as a result of vaccination or COVID-19 infection, and placental transfer of COVID-19 vaccine-induced immunity, revealed that the vaccinated subcohort (66.6% of respondents) has a moderate (mean: 78.8%; range: 72.7–88.1%) while the unvaccinated (33.4% of respondents) subcohort has a poor (mean 40.0%; range: 24.5–62.0%) knowledge level in that respect (Table 3 and Table 4). Identified by us, differences in knowledge level in the field of COVID-19 vaccination between vaccinated and unvaccinated women are in line with results presented by Duong and coworkers [47]. The lack of or poor knowledge level concerning the impact of COVID-19 vaccination on women during and after pregnancy reveals educational gaps that must be filled in this group of respondents.

In the group of women who had not chosen to be vaccinated, the fear of possible post-vaccination complications was higher than the fear of infection caused by the SARS-CoV-2 virus. In line with the above, it is important that the obstetric staff must be constantly educated in the field of the COVID-19 vaccination to be well prepared to impart reliable knowledge to women of reproductive age who are expecting a baby or are already mothers [44]. Furthermore, as pointed out previously [48], the role of experts in the reliable delivery of information on COVID-19 vaccines and also question-and-answer sessions by different media channels should be substantially strengthened since a high acceptance of vaccination is warranted by a good information policy [44].

The women’s knowledge concerning COVID-19 vaccination was related to sociodemographic and very few obstetric variables (Supplementary Materials Tables S1 and S2). The lower level of women’s knowledge in the field of COVID-19 vaccination and practices towards COVID-19 is associated with younger age, lower education level, and single motherhood (Supplementary Materials Table S1) and overlaps with factors identified previously by Saeed and coworkers [49]. Among obstetric data, the mode of delivery was associated with answers to some questions concerning COVID infection and vaccination (Supplementary Materials Table S2).

The assessment of women’s knowledge level in the field of COVID-19 vaccination among women during and after pregnancy revealed significant differences in relation to the obstetric state in the vaccinated but not the unvaccinated cohort (Table 5). For the vaccinated cohort, the highest level of anxiety against SARS-CoV-2 infection during pregnancy was declared only by 29.3% of pregnant women, while in the cohort of women after delivery, it was reported by 59.3% of respondents (*p* < 0.001). The low level of fear revealed in our study clearly indicates educational gaps in the subgroup of women of reproductive age, which require emergency intervention. Pregnant women should be aware that due to the pregnancy-related physiological changes, they are in the group at higher risk of respiratory complications related to viral infections, including COVID-19 [13,14,15,16,17].

On the other hand, a moderate level of knowledge has been found for issues concerning possible transmission of the virus and transfer of specific anti-SARS-CoV-2 antibodies generated as a result of vaccination or COVID-19 infection via breast milk; however, for women after delivery, the declared knowledge in this field was significantly higher than for pregnant women, namely 79.0% and 64.7%, respectively. The higher knowledge level declared by women after delivery concerning the health benefits of breastfeeding is understandable; however, due to the appearance of new coronavirus variants that are more dangerous for children under two years old, the women’s awareness should be higher and focused on delivering specific antibodies along with milk to prevent this disturbing phenomenon.

During pregnancy, women must take care of their own and their offspring’s health. Such a mindset was clearly reflected in the results of our survey, namely, a willingness to protect a child reported by mothers was at a comparable level as self-protection against a severe course of COVID-19. The primary reasons for getting a COVID-19 vaccine named by pregnant women included protection of oneself during the perinatal period (62.1%), protection against a severe course of the disease (60.3%), and protection of the developing fetus (54.0%). Similarly, mothers who have already given birth identified the main reasons for receiving vaccination as protection against a severe course of the disease (62.4%) and protection of newborns (57.2%). These reasons indicated by the women in our study during the fourth wave of the pandemic for making the decision to get vaccinated are in line with those indicated in a study conducted at the beginning of the pandemic on a group of Polish adults, namely the protection of themself (64.2%) and relatives (60.6%) from infection [7].

A small fraction of the respondents, namely 4.0% of pregnant women and 4.5% of mothers, indicated as reasons to get vaccinated willingness to receive a digital COVID-19 certification (vaccine passport) and related benefits (e.g., better medical care and the possibility of hospitalization with their child). Nevertheless, despite the occurrence of successive waves of the pandemic, the attitude and the main reasons for willingness to vaccinate against COVID-19 of the Polish population have not substantially changed.

Among vaccinated respondents, the largest group constituted women who have received vaccines based on messenger RNA (mRNA) technology produced by Pfizer/BioNTech: 77.7% of pregnant women and 73.6% of women who had already given birth, while the vaccines provided by Moderna were received by 10.3% and 13.0% of women during and after pregnancy, respectively. These results are in line with previously reported preferences and the highest level of trust for mRNA vaccines in the Polish adult population at the beginning of vaccine implementation [7]; however, it must be taken into consideration that the Pfizer/BioNTech vaccine was the first one approved for pregnant and lactating women [9].

Women’s lives during pregnancy and postpartum involve significant changes, not only physiological but also psychosocial [50], which may be additionally affected by the uncertain epidemiological situation related to the COVID-19 pandemic [51,52]. During the first three months of the pandemic, postnatal mental distress seemed to be associated more with the change in obstetric status and other personal factors than with the COVID-19 pandemic restrictions [53]; however, with the progression of the pandemic, the adverse impact of COVID-19 on the mental health of women in the perinatal period has been documented [51]. The frequency of occurrence of mental disorders, such as depression and anxiety, including thoughts of self-harm [52], increased [54,55,56], and unfortunately, it translated into the psychological and developmental disturbances of their children. So far, the fear of COVID-19 vaccination among women in the perinatal period has not been assessed in Poland. In our study, the majority of unvaccinated respondents, namely 73.2% of pregnant women and 69.8% of mothers, among stated reasons to avoid COVID-19 vaccination, declared possible post-vaccination complications. Especially high concerns about COVID-19 vaccines included the adverse impact of the vaccine on their own (61.0% of pregnant women and 60.7% of mothers) and child’s health (81.7% of pregnant women and 55.7% of mothers). The main factors indicated by women in our analyzed cohort, albeit with varying frequency, were in line with those reported previously [7,15,44].

It should be emphasized that the lack of COVID-19 vaccination recommendations for pregnant and breastfeeding women at the beginning of the implementation of the global strategy of coronavirus vaccine had a crucial influence on the decision not to accept the vaccine against SARS-CoV-2. Our results clearly indicate that despite the introduction of COVID-19 vaccination recommendations for pregnant and breastfeeding women [9,10,11], the main concerns of women have still not been overcome. Despite unlimited access to COVID-19 vaccines in Poland, the level of vaccination is still one of the lowest in Europe, clearly confirming that not availability but vaccine hesitancy is the main limiting factor. Hirshberg and coworkers [57] drew similar conclusions based on a USA pilot study conducted during the second quarter of 2021 on high-risk obstetrical patients. On the other hand, when the respondents had the opportunity to indicate the reason for not getting vaccinated, one of those most frequently self-reported was the lack of or limited studies confirming the safety of the COVID-19 vaccine (6.3% of mothers and 2.4% of pregnant women) (Supplementary Materials Figure S2). Our findings clearly show that the Polish population needs intervention via deep educational programs aimed at imparting reliable knowledge about mRNA technology and COVID-19 vaccination [58], although such vaccine technology has been known for 20 years and confirmed as safe [45,59]. Moreover, as stated by world authorities, COVID-19 vaccination during pregnancy outweighs any possible risks for pregnant women and their offspring and additionally delivers immune protection for newborns of vaccinated mothers [60,61,62,63,64,65,66]. Recent research indicates [60,61,62,63,64,65,66] that mRNA COVID-19 vaccination has no impact on pregnant women and their babies, namely regarding the risk of miscarriages, preterm births, or side effects in unborn babies, among others.

The first vaccines were available at the end of 2020 [9], and their appearance induced a high concern about their safety and effectiveness; however, such a state of affairs also accompanied the recommendation concerning influenza vaccination during pregnancy in the past [67]. In fact, the morbidity and mortality of COVID-19 during the perinatal period are closely related to the availability and acceptance of COVID-19 vaccination [9]; however, despite easy access to such protection, the declared level of vaccination is not satisfactory and requires urgent educational action. Knowledge of these areas translates into dedicated activities that can change the attitude towards vaccination in the target group of women of reproductive age.

As shown by the results of our analysis, despite the detailed data with regard to COVID-19 vaccination based on evidence-based medicine, this knowledge is largely ignored in Poland; therefore, action should be taken to identify areas that require urgent intervention. The multi-logistic regression analysis revealed that unmarried women living in small cities and rural areas belong to the group that needs special attention. Moreover, a declaration of a low level of fear of SARS-CoV-2 infection and the lack of knowledge in the fields of COVID-19 vaccine-induced immune protection of the fetus (placental transfer) and newborns are indicated as the main predictors associated with avoidance of COVID-19 vaccination by the population of Polish women in reproductive age during the fourth wave of the pandemic. The sociodemographic variables identified in our study are in line with data provided by Stuckelberger and coworkers [40].

In this difficult and potentially dangerous situation for the population’s health, it seems that a solution that may bring an increase in awareness and thus translate into increased vaccination is the implementation of interventional educational campaigns. The high levels of anxiety among pregnant women and mothers about infection caused by the SARS-CoV-2 virus might be decreased by providing dedicated education and encouraging vaccination. For this reason, raising awareness in the population of women in the perinatal period and emphasizing that women who vaccinate themselves, in fact, protect their offspring due to the enhancement of their immune system by providing the set of specific antibodies against SARS-CoV-2 via placental transfer and with breast milk, are extremely important.

### Strengths and Limitations of Our Study

To the best of our knowledge, this is the first study in Poland that investigates the COVID-19 vaccination level and attitude among women during pregnancy and after giving birth, covering an important field of public health, since the level of vaccination in Poland remains at one of the lowest levels among European countries. The analyzed cohort size, as well as the demographic data, suggest that the obtained results are representative of Polish women of reproductive age; however, we consider as a limitation of our study the impossibility of comparing our data with the period when the first recommendations regarding vaccination of pregnant women appeared, as no such data are available. Another limitation that warrants a comment here is the comparison of our data with the statistics published by the other countries due to the differences in restrictions introduced as well as shifts in the dynamics of development of successive waves of the COVID-19 pandemic.

Nevertheless, it should be noted that the use of an online survey may have an impact on the sample towards those more familiar with using technology and, therefore, may not be representative of some class of society who do not have access to the Internet and social media; however, according to the Polish Central Statistical Office, in 2021 [68], 92.4% of households had Internet access. In fact, the lack of access to the Internet and social media mainly concerns people above 60 years who were not included in the study group. Additionally, in our study, more than three fourth of respondents had a university education, while according to the Overview of the educational system (EAG 2021) [69], in Poland, 53% of 25–34-year-old women had a tertiary qualification in 2020. In light of the above, this survey included women who, in our opinion, were looking for information not only about the perinatal period but also about the benefits of vaccination; therefore, the analyzed cohort did not fully reflect the general population of Polish women and should be pointed out as a limitation of our survey.

In the near future, it is worth evaluating whether there is progress in accepting the vaccine when there is substantially greater access to additional and confirmed scientific data, particularly when new variants of the coronavirus are identified as more dangerous for children under two years compared to the beginning of the pandemic. Emerging reliable results of research on larger analyzed cohorts and intensive information and promotion activities undertaken over the world by the numerous societies and committees as well as governmental institutions regarding the popularization of vaccinations bring the assumed effect, i.e., an increase in vaccination of Polish women in reproductive age.

## 5. Conclusions

The results of our research clearly indicate that dedicated knowledge should be provided for specific target groups. For women of reproductive age, one of the most important tasks must be efficiently delivering information that confirms the safety of the COVID-19 vaccination not only for pregnant women but also for the developing fetus. Moreover, an insufficiently accentuated aspect of the vaccination for pregnant and breastfeeding women is the protection of their offspring due to the transport of SARS-CoV-2-specific antibodies by the placenta and transfer with breast milk, which translates into indisputable health benefits for new generations. The dedicated information policy, especially in the group of mothers, is particularly important also due to the decisive role in terms of vaccination of their offspring.

In line with the general knowledge in this field, we found that declarations of a high anxiety level as regards SARS-CoV-2 infection translate into the increased willingness of women to be vaccinated and, finally, on the wellbeing of women of reproductive age. In the light of the obtained data, the overriding goal should be to strengthen the national strategy to support and promote vaccination among Polish women of reproductive age, especially since the successive variants of the SARS-Co-2 virus are responsible for the increase in the incidence of infections and an increase in the severity of the disease and post-COVID-19 complications occurring with much higher frequency in the group of children under two years old.

## Figures and Tables

**Table 1:** Participants’ characteristics.

Data		n/N 1196	%
**Age** **(years)**	18–25	131/1196	11.0
	26–30	461/1196	38.5
	31–35	422/1196	35.3
	36–40	164/1196	13.7
	≥41	18/1196	1.5
**Pre-pregnancy BMI** **(kg/m^2^)**	underweight (<18.5)	84/1196	7.0
	normal weight (18.5–24.9)	765/1196	64.0
	overweight (25–29.9)	219/1196	18.3
	obesity class 1 (30–34.9)	101/1196	8.4
	obesity class 2 (35–39.9)	26/1196	2.2
	obesity class 3 (≥40)	1/1196	0.1
**Residence**	urban, above 100,000 residents	632/1196	52.8
	urban, 10,000–100,000 residents	226/1196	18.9
	urban, <10,000 residents	76/1196	6.4
	rural	262/1196	21.9
**Education**	vocational and primary	30/1196	2.5
	high school	223/1196	18.7
	university	943/1196	78.8
**Marital status**	married	959/1196	80.2
	cohabiting	214/1196	17.9
	single parent and divorced	23/1196	1.9
**Currently obstetric** **state**	pregnant women in 1 trimester	16/1196	1.3
	pregnant women in 2 trimester	64/1196	5.4
	pregnant women in 3 trimester	176/1196	14.7
	women after delivery	940/1196	78.6
**Mode of delivery**	vaginal birth	528/940	56.2
	elective cesarean section	215/940	22.8
	emergency cesarean section	197/940	21.0
**Physiological pregnancy**	Yes	993/1196	83.0
	No	203/1196	17.0
**Difficulties in conceiving** **a child**	Yes	278/1196	23.2
	No	918/1196	76.8
**Medically assisted** **procreation**	Yes	93/1196	7.8
	No	1103/1196	92.2
**Miscarriage in the past**	Yes	259/1196	21.7
	No	937/1196	78.3

The table shows the percentage of respondents in the given subgroup (n) in relation to all respondents (N) for whom the specific information was available.

**Table 2:** Level of COVID-19 vaccination among women in relation to sociodemographic characteristics.

Sociodemographic Data		Vaccinated N = 796 % (n)	Unvaccinated N = 400 % (n)	χ^2^ Test	*p*-Value
**Age (years)**	18–25	7.9% (63)	17.0% (68)	35.95	<0.001
	26–30	36.7% (292)	42.2% (169)		
	31–35	38.8% (309)	28.3% (113)		
	36–40	14.6% (116)	12.0% (48)		
	≥41	2.0% (16)	0.5% (2)		
**Pre-pregnancy BMI, kg/m^2^**	underweight (<18.5)	6.7% (53)	7.8% (31)	1.06	0.95
	normal weight (18.5–24.9)	64.2% (511)	63.5% (254)		
	overweight (25–29.9)	18.3% (146)	18.2% (73)		
	obesity class 1 (30–34.9)	8.4% (67)	8.5% (34)		
	obesity class 2 (35–39.9)	2.3% (18)	2.0% (8)		
	obesity class 3 (≥40)	0.1% (1)	0% (0)		
**Residence**	urban, above 100,000 residents	60.0% (478)	38.5% (154)	55.17	<0.001
	urban, 10,000–100,000 residents	17.6% (140)	21.5% (86)		
	urban, <10,000 residents	4.8% (38)	9.5% (38)		
	rural	17.6% (140)	30.5% (122)		
**Education**	vocational and primary	1.6% (13)	4.3% (17)	48.682	<0.001
	high school	13.7% (109)	28.5% (114)		
	university	84.7% (674)	67.2% (269)		
**Marital status**	married	84.1% (669)	72.5% (290)	22.36	<0.001
	cohabiting	14.4% (115)	24.8% (99)		
	single parent and divorced	1.5% (12)	2.7% (11)		
**Current obstetric state**	pregnant women 1 trimester	1.0% (8)	2.0% (8)	3.27	0.35
	pregnant women 2 trimester	5.3% (42)	5.5% (22)		
	pregnant women 3 trimester	15.6% (124)	13.0% (52)		
	women after delivery	78.2% (622)	79.6% (318)		
**Type of delivery**	vaginal birth	53.9% (335)	60.7% (193)	4.32	0.11
	elective cesarean section	23.6% (147)	21.4% (68)		
	emergency cesarean section	22.5% (140)	17.9% (57)		
**Physiological pregnancy**	Yes	82.7% (658)	83.8% (335)	0.22	0.63
	No	17.3% (138)	16.2% (65)		
**Difficulties in conceiving a child**	Yes	23.7% (189)	22.3% (89)	0.33	0.56
	No	48.5% (607)	77.8% (311)		
**Medically assisted** **procreation**	Yes	8.3% (66)	6.7% (27)	0.88	0.34
	No	91.7% (730)	93.3% (373)		
**Miscarriage in the past**	Yes	22.0% (175)	21.0% (84)	0.20	0.69
	No	78.0% (621)	79.0% (316)		

The table shows the percentage of respondents in the given subgroup (n) in relation to all respondents (N) for whom the specific information was available.

**Table 3:** Level of COVID-19 vaccination among pregnant and breastfeeding women in relation to sociodemographic and obstetric characteristics.

Sociodemographic Data		Pregnant Women		Women after Delivery		Vaccinated Women, Pregnant vs. after Delivery		Unvaccinated Women, Pregnant vs. after Delivery	
		Vaccinated N = 174 % (n)	Unvaccinated N = 82 % (n)	Vaccinated N = 622 % (n)	Unvaccinated N = 318 % (n)	χ^2^ Test	*p*-Value	χ^2^ Test	*p*-Value
**Age (years)**	18–25	12.6% (22)	18.3% (15)	6.6% (41)	16.7% (53)	9.17	0.057	3.87	0.42
	26–30	36.2% (63)	40.2% (33)	36.8% (229)	42.8% (136)				
	31–35	39.1% (68)	34.2% (28)	38.7% (241)	26.7% (85)				
	36–40	10.9% (19)	7.3% (6)	15.6% (97)	13.2% (42)				
	≥41	1.2% (2)	0.0% (0)	2.3% (14)	0.6% (2)				
**Pre-pregnancy BMI (kg/m^2^)**	underweight (<18.5)	4.6% (8)	11.0% (9)	7.2% (45)	6.9% (22)	6.96	0.22	22.61	<0.001
	normal weight (18.5–24.9)	66.1% (115)	53.6% (44)	63.7% (396)	66.1% (210)				
	overweight (25–29.9)	20.1% (35)	12.2% (10)	17.8% (111)	19.8% (63)				
	obesity class 1 (30–34.9)	7.5% (13)	17.1% (14)	8.7% (54)	6.3% (20)				
	obesity class 2 (35–39.9)	1.2% (2)	6.1% (5)	2.6% (16)	0.9% (3)				
	obesity class 3 (≥40)	0.6% (1)	0.0% (0)	0.0% (0)	0.0% (0)				
**Residence**	urban, above 100,000 residents	71.8% (125)	57.3% (47)	56.8% (353)	33.7% (107)	13.93	<0.005	18.5	<0.001
	urban, 10,000–100,000 residents	10.3% (18)	18.3% (15)	19.6% (122)	22.3% (71)				
	urban, <10,000 residents	3.4% (6)	9.8% (8)	5.1% (32)	9.4% (30)				
	rural	14.4% (25)	14.6% (12)	18.5% (115)	34.6% (110)				
**Education**	vocational and primary	1.7% (3)	3.7% (3)	1.6% (10)	4.4% (14)	0.50	0.77	0.11	0.94
	high school	12.1% (21)	29.2% (24)	14.2% (88)	28.3% (90)				
	university	86.2% (150)	67.1% (55)	84.2% (524)	67.3% (214)				
**Marital status**	married	79.9% (139)	61.0% (50)	85.2% (530)	75.5% (240)	3.13	0.20	2.37	0.12
	cohabiting	17.8% (31)	37.8% (31)	13.5% (84)	21.4% (68)				
	single parent and divorced	2.3% (4)	1.2% (1)	1.1% (8)	0.6% (10)				
**Physiological pregnancy**	Yes	89.7% (156)	89.0% (73)	80.7% (502)	82.4% (262)	7.60	<0.006	2.11	0.14
	No	10.3% (18)	11.0% (9)	19.3% (120)	17.6% (56)				
**Difficulties in conceiving a child**	Yes	22.4% (39)	25.6% (21)	24.1% (150)	21.4% (68)	0.22	0.64	0.67	0.41
	No	77.6% (135)	74.4% (61)	75.9% (472)	78.6% (250)				
**Medically assisted procreation**	Yes	18.0% (14)	3.7% (3)	8.4% (52)	7.5% (24)	0.02	0.89	1.56	0.21
	No	92.0% (160)	96.3% (79)	91.6% (570)	92.5% (294)				
**Miscarriage in the past**	Yes	21.8% (38)	23.2% (19)	22.0% (137)	20.4% (65)	0.003	0.95	0.29	0.58
	No	78.2% (136)	76.8% (63)	78.0% (485)	79.6% (253)				

The table shows the percentage of respondents in the given subgroup (n) in relation to all respondents (N) for whom the specific information was available.

**Table 4:** Level of COVID-19 knowledge in relation to COVID-19 vaccination among women.

COVID-19 Knowledge Level		Vaccinated N = 796 % (n)	Unvaccinated N = 400 % (n)	χ^2^ Test	*p*-Value
Were you afraid of SARS-CoV-2 infection during pregnancy/lactation?	1	4.9% (39)	21.5% (86)	197.04	<0.001
	2	3.6% (29)	14.3% (57)		
	3	14.7% (117)	25.5% (102)		
	4	21.5% (171)	16.3% (65)		
	5	55.3% (440)	22.5% (90)		
Did you suffer from COVID-19 during pregnancy?	Yes	11.2% (89)	15.3% (61)	45.94	<0.001
	No	79.8% (635)	63.0% (252)		
	I do not know	9.0% (72)	21.8% (87)		
Is SARS-CoV-2 transmitted through breastfeeding?	Yes	4.5% (36)	7.5% (30)	22.75	<0.001
	No	75.3% (599)	62.0% (248)		
	I do not know	20.2% (161)	30.5% (122)		
Can breastfeeding after the mother has COVID-19 protect the baby from SARS-CoV-2 infection?	Yes	72.7% (579)	45.0% (180)	92.32	<0.001
	No	8.7% (69)	22.3% (89)		
	I do not know	18.6% (148)	32.8% (131)		
Do you think that immunity achieved after COVID-19 vaccination might provide immune protection to the fetus and newborn (placental transfer)?	Yes	88.1% (701)	28.5% (114)	449.63	<0.001
	No	2.0% (16)	28.8% (115)		
	I do not know	9.9% (79)	42.8% (171)		
Do you think that immunity achieved after COVID-19 vaccination might be transferred with human milk to the newborns/infants?	Yes	79.6% (634)	24.5% (98)	346.43	<0.001
	No	6.8% (54)	33.5% (134)		
	I do not know	13.6% (108)	42.0% (168)		

The table shows the percentage of respondents in the given subgroup (n) in relation to all respondents (N) for whom the specific information was available. Definitely not afraid—1 point on a five-point scale of fear. Definitely afraid—5 points on a five-point scale of fear.

**Table 5:** Level of COVID-19 knowledge in relation to COVID-19 vaccination among women during pregnancy and after delivery.

		Pregnant Women		Women after Delivery		Vaccinated Women, Pregnant vs. after Delivery		Unvaccinated Women, Pregnant vs. after Delivery	
		Vaccinated N = 174 % (n)	Unvaccinated N = 82 % (n)	Vaccinated N = 622 % (n)	Unvaccinated N = 318 % (n)	χ^2^ Test	*p*-Value	χ^2^ Test	*p*-Value
Were you afraid of SARS-CoV-2 infection during pregnancy/lactation?	1	4.6% (8)	23.2% (19)	5.0% (31)	21.1% (67)	37.22	<0.001	2.91	0.57
	2	4.6% (8)	18.3% (15)	3.4% (21)	13.2% (42)				
	3	18.4% (32)	20.7% (17)	13.7% (85)	26.7% (85)				
	4	31.6% (55)	18.3% (15)	18.6% (116)	15.7% (50)				
	5	29.3% (51)	19.5% (16)	59.3% (369)	23.3% (74)				
Did you suffer from COVID-19 during pregnancy?	Yes	7.5% (13)	12.2% (10)	12.2% (76)	16.0% (51)	3.12	0.21	1.89	0.39
	No	82.8% (144)	69.5% (57)	78.9% (491)	61.3% (195)				
	I do not know	9.8% (17)	18.3% (15)	8.8% (55)	22.6% (72)				
Is SARS-CoV-2 transmitted through breastfeeding?	Yes	9.8% (17)	7.3% (6)	3.1% (19)	7.5% (24)	28.72	<0.001	0.07	0.96
	No	60.9% (106)	61.0% (50)	79.3% (493)	62.3% (198)				
	I do not know	29.3% (51)	31.7% (26)	17.7% (110)	30.2% (96)				
Can breastfeeding after the mother has COVID-19 protect the baby from SARS-CoV-2 infection?	Yes	64.9% (113)	37.8% (31)	74.9% (466)	46.9% (149)	64.03	<0.001	2.47	0.29
	No	13.2% (23)	23.2% (19)	7.4% (460	22.0% (70)				
	I do not know	21.8% (38)	39.0% (32)	17.7% (110)	31.1% (99)				
Do you think that immunity achieved after COVID-19 vaccination might provide immune protection to the fetus and newborn (placental transfer)?	Yes	92.5% (161)	32.9% (27)	86.8% (540)	27.4% (87)	4.51	0.10	1.06	0.59
	No	1.7% (3)	28.0% (23)	2.1% (13)	28.9% (92)				
	I do not know	5.7% (10)	39.0% (32)	11.1% (69)	43.7% (139)				
Do you think that immunity achieved after COVID-19 vaccination might be transferred with human milk to the newborns/infants?	Yes	68.4% (119)	26.8% (22)	82.8% (515)	23.9% (76)	17.48	<0.001	0.77	0.68
	No	10.9% (19)	35.4% (29)	5.6% (35)	33.0% (105)				
	I do not know	20.7% (36)	37.8% (31)	11.6% (72)	43.1% (137)				

The table shows the percentage of respondents in the given subgroup (n) in relation to all respondents (N) for whom the specific information was available.

**Table 6:** Predictors of a lack of COVID-19 vaccination among women.

Data	Odds Ratio (OR) (95% Lower—Upper Confidence Interval (CI))	*p*-Value
**Age (years)**	0.96 (0.92–1.00)	<0.06
**Residence** urban, above 100,000 residents (ref) urban, 10,000–100,000 residents urban, <10,000 residents rural	1.52 (0.98–2.37) 2.06 (1.05–4.02) 1.89 (1.25–2.87)	0.06 <0.04 <0.003
**Marital status** married (ref) cohabiting single parent & divorced	1.76 (1.15–2.70) 1.00 (0.34–2.94)	<0.009 0.99
**Were you afraid of SARS-CoV-2 infection during pregnancy/lactation?** **5** (I was DEFINITELY AFRAID) **(ref)** **4** **3** **2** **1** (I was DEFINITELY NOT AFRAID)	1.90 (1.20–3.02) 4.31 (2.75–6.74) 7.21 (3.84–13.54) 5.84 (3.32–10.27)	<0.007 <0.001 <0.001 <0.001
**Did you suffer from COVID-19 during your pregnancy?** No (ref) Yes I do not know	2.32 (1.43–3.77) 2.67 (1.66–4.28)	<0.001 <0.001
**Is SARS-CoV-2 transmitted through breastfeeding?** No (ref) Yes I do not know	2.32 (1.15–4.70) 1.24 (0.82–1.88)	<0.02 0.3
**Does breastfeeding protect children from COVID-19?** Yes (ref) No I do not know	1.86 (1.13–3.07) 1.08 (0.70–1.67)	<0.02 0.73
**Do you think that immunity achieved after COVID-19 vaccination might provide immune protection to the fetus and newborn (placental transfer)?** Yes (ref) No I do not know	23.86 (12.99–43.84) 10.77 (7.20–16.10)	<0.001 <0.001

ref—reference category.

## Data Availability

The data presented in this study are available on request from the corresponding author.

## Supplementary Materials

The following supporting information can be downloaded at: https://www.mdpi.com/article/10.3390/ijerph19116872/s1, Table S1: Level of COVID-19 knowledge in relation to sociodemographic variables; Table S2: Level of COVID-19 knowledge in relation to obstetric variables; Figure S1: Respondents’ reasons for COVID-19 vaccination; Figure S2: Reasons to avoid COVID-19 vaccination declared by analyzed cohort of respondents. The Questionnaire is available online at https://forms.gle/ETxdQEWUvTfoC5VR9 (accessed on 31 May 2022).
