# Supplementary material for: Attitude and Level of COVID-19 Vaccination among Women in Reproductive Age during the Fourth Pandemic Wave: A Cross-Sectional Study in Poland
Source: Int J Environ Res Public Health. 2022 Jun 4;19(11):6872. doi: 10.3390/ijerph19116872 (PMC9180577; doi:10.3390/ijerph19116872)
Supplement: Supplementary file 1 [file ijerph-19-06872-s001.zip › ijerph-1635931-supplementary.pdf]

Supplementary Materials

Table S1. Level of COVID-19 knowledge in relation to sociodemographic variables.

	Sociodemographic data				
	Continuous Data[a]		Categorical Data[b]		
	**Age**	**BMI**	**Residence[A]**	**Education[B]**	**Marital Status[C]**
Were you afraid of SARS-CoV-2 infection during pregnancy/lactation?	p = 0.0007	p =0.7	χ^2 15.59 p = 0.21	χ^2 37.32 p = 0.00001	χ^2 23.88 p = 0.002
Did you suffer from COVID-19 during your pregnancy?	p =0.1	p =0.7	χ^2 18.33 p = 0.005	χ^2 11.40 p = 0.02	χ^2 3.72 p = 0.44
Is SARS-CoV-2 transmitted through breastfeeding?	p = 0.007	p =0.3	χ^2 21.29 p = 0.001	χ^2 20.92 p = 0.0003	χ^2 17.33 p = 0.001
Does breastfeeding protect children from COVID-19?	p = 0.03	p =0.7	χ^2 16.04 p = 0.01	χ^2 26.11 p = 0.00003	χ^2 7.26 p = 0.12
Do you think that immunity achieved after COVID-19 vaccination might provide immune protection to the fetus and newborn (placental transfer)?	p = 0.001	p =0.6	χ^2 60.92 p = 0.00001	χ^2 74.46 p = 0.00001	χ^2 12.91 p = 0.01
Do you think that immunity achieved after COVID-19 vaccination might be transferred with human milk to the newborns/infants?	p = 0.1	p =0.4	χ^2 14.56 p = 0.02	χ^2 34.04 p = 0.00001	χ^2 15.80 p = 0.003

[a] the Kruskal–Wallis test was used to evaluate the differences between the groups

[b] the chi-squared test was used to evaluate the differences between the groups

[A] place of the residence:>100,000 inhabitants, 10,000–100,000 inhabitants, <10,000 inhabitants and rural area

[B] education level: university, high school, primary and vocational

[C] marital status: married, cohabiting, single parent and divorced

Table S2. Level of COVID-19 knowledge in relation to obstetric variables.

	Obstetric data[a]					
	**Current obstetric state[A]**	**Type of delivery[B]**	**Physiological pregnancy[C]**	**Difficulties in conceiving a child[D]**	**Medically assisted procreation[E]**	**Miscarriage in the past[F]**
Were you afraid of SARS-CoV-2 infection during pregnancy/lactation?	χ^2 9.16 p = 0.68	χ^2 41.56 p = 0.00004	χ^2 3.20 p = 0.52	χ^2 1.22 p = 0.87	χ^2 4.66 p = 0.32	χ^2 3.13 p = 0.53
Did you suffer from COVID-19 during your pregnancy?	χ^2 7.36 p = 0.28	χ^2 14.17 p = 0.02	χ^2 5.60 p = 0.06	χ^2 7.49 p = 0.02	χ^2 4.28 p = 0.11	χ^2 6.31 p = 0.04
Is SARS-CoV-2 transmitted through breastfeeding?	χ^2 3.08 p = 0.79	χ^2 23.28 p = 0.0007	χ^2 3.84 p = 0.14	χ^2 1.93 p = 0.38	χ^2 0.17 p = 0.91	χ^2 2.44 p = 0.29
Does breastfeeding protect children from COVID-19?	χ^2 5.82 p = 0.44	χ^2 11.59 p = 0.07	χ^2 4.60 p = 0.10	χ^2 0.17 p = 0.91	χ^2 0.73 p = 0.69	χ^2 2.25 p = 0.32
Do you think that immunity achieved after COVID-19 vaccination might provide immune protection to the fetus and newborn (placental transfer)?	χ^2 9.74 p = 0.13	χ^2 13.25 p = 0.03	χ^2 1.69 p = 0.42	χ^2 0.26 p = 0.87	χ^2 1.91 p = 0.38	χ^2 2.61 p = 0.27
Do you think that immunity achieved after COVID-19 vaccination might be transferred with human milk to the newborns/infants?	χ^2 7.92 p = 0.24	χ^2 12.42 p = 0.05	χ^2 6.57 p = 0.03	χ^2 1.47 p = 0.47	χ^2 1.69 p = 0.42	χ^2 7.09 p = 0.02

[a] the chi-squared test was used to evaluate the differences between the groups
[A] current obstetric state: pregnancy, a woman who gave birth to a child
[B] type of delivery: vaginal birth, elective or emergency caesarean section
[C] physiological pregnancy: with or without complications
[E] difficulties in conceiving a child: yes, no
[F] miscarriage in the past: yes, no

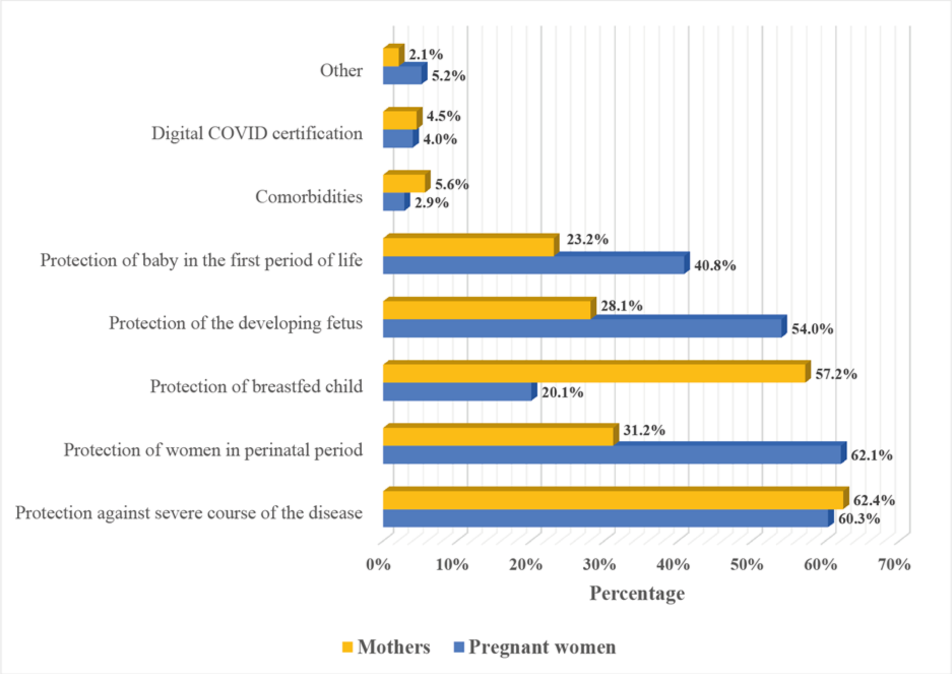

Figure S1. Respondents' reasons for COVID-19 vaccination.

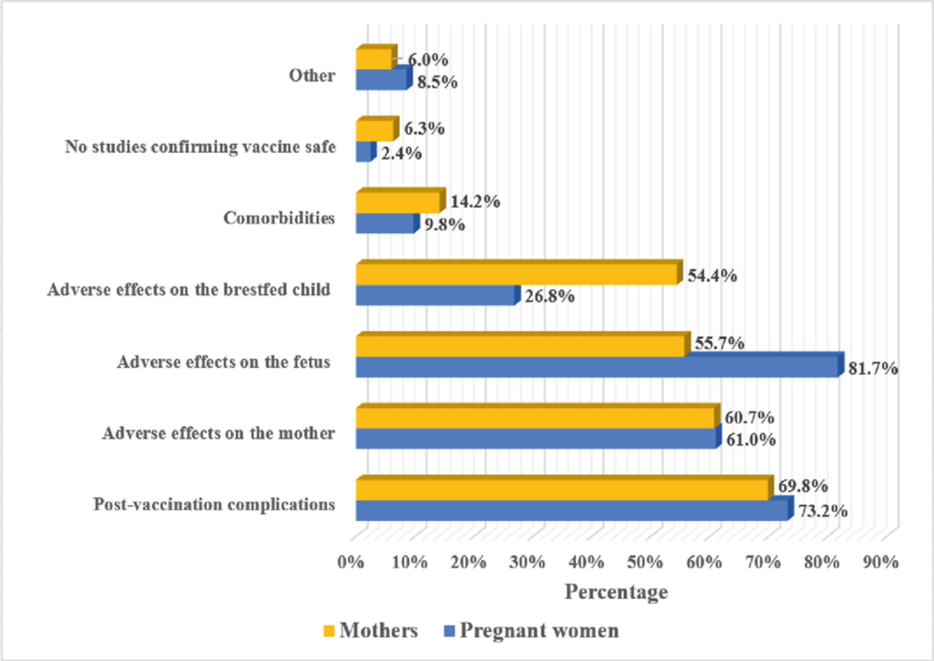

Figure S2. Reasons to avoid COVID-19 vaccination declared by analyzed cohort of respondents.
